# Monoclonal antibody treatment of COVID-19 in a pregnant woman with common variable immunodeficiency

**DOI:** 10.1186/s13223-022-00730-x

**Published:** 2022-10-16

**Authors:** Babak Aberumand, Ramy Kamal, Brock McKinney, Stephen Betschel

**Affiliations:** 1 Division of Allergy and Immunology, Department of Medicine, St. Michael’s Hospital, 30 Bond St, Toronto, ON M5B 1W8 Canada; 2 Divisions of Family and Emergency Medicine, Orillia Soldiers’ Memorial Hospital, Toronto, Canada; 3 Division of Obstetrics and Gynecology, Orillia Soldiers Memorial Hospital, Toronto, ON Canada

**Keywords:** COVID-19, SARS-CoV-2, CVID, Common variable immunodeficiency, Pregnancy, Sotrovimab, Xevudy, Monoclonal antibodies, Primary immunodeficiency, Inborn errors of immunity

## Abstract

**Background:**

Since the first reported case of COVID-19, infections due to the virus have ranged from mild to severe. Patients with inborn errors of immunity are thought to be at increased risk for infections such as COVID-19 due to the nature of their disease and being immunocompromised. Similarly, pregnant women by nature of physiological changes in immunity are susceptible to infections and consequently are felt to be at greater risk of contracting COVID-19 with potential grave consequences for not only the mother but also the fetus. Early treatment with novel therapeutics against the SARS-CoV-2 virus to prevent progression and these complications is paramount.

**Case Presentation:**

A 31-year-old woman with a 22-year history of common variable immunodeficiency on subcutaneous immunoglobulin replacement therapy and 24 weeks pregnant with her third child presented to the Emergency Department with two-day history of pharyngitis that progressed to include nasal and chest congestion, non-productive cough and shortness of breath. Her vitals indicated temperature of 35 degrees Celsius, heart rate of 109 beats per minute, blood pressure 142/92 mmHg, respiratory rate 22/min and an oxygen saturation of 99% on room air. A workup was done and she was found to be positive for SARS-CoV-2 virus confirmed by PCR. She had a close contact, her husband, who had tested positive a few days prior. She had been previously vaccinated with three doses of the Moderna COVID-19 (Spikevax ®) vaccine. As she met the criteria for monoclonal antibody treatment, she received Sotrovimab on the same day of testing positive and tolerated it well with no side-effects. Her symptoms resolved within two to three days.

**Conclusion:**

Our case, is the first to our knowledge, of a pregnant patient with common variable immunodeficiency diagnosed with COVID-19 and symptomatic successfully receiving treatment with Sotrovimab. Her rapid resolution of symptoms makes the use of monoclonal antibodies such as Sotrovimab a safe and useful option in this unique population.

## Background

Since the first reported case of SARS-CoV-2 (COVID-19), much work has been conducted on identifying diseases and comorbidities associated with a higher risk of contracting the virus and risk factors for developing a more severe course upon infection. This high-risk population includes a group of heterogenous genetic disorders with impairment in various components of the immune system, known collectively as inborn errors of immunity (IEI), formerly known as primary immunodeficiency disorders [1]. Patients with IEI are at increased risk for infections such as SARS-CoV-2, by virtue of the nature of their disease and being immunocompromised [2]. Similarly, pregnant women by nature of physiological changes in immunity are susceptible to infections, in particular respiratory, and consequently are felt to be at greater risk of contracting SARS-CoV-2. Should they contract SARS-CoV-2, pregnant women are more likely to be admitted to the intensive care unit (ICU), require invasive ventilation, receive extracorporeal membrane oxygenation and die than non-pregnant women of reproductive age [3]. Furthermore, pregnant women with SARS-CoV-2 infection are more likely to deliver preterm and are at a higher risk of maternal death compared to pregnant women without SARS-CoV-2 infection [3]. Despite the potential for disease progression and severe outcomes with SARS-CoV-2, treatment options and their efficacy in these populations, at this time, are limited.

## Case presentation

We present a case of monoclonal antibody, sotrovimab, treatment in a COVID-19 positive pregnant patient with mild to moderate symptoms and an underlying IEI. Our patient was a 24-week pregnant G3P2 31-year-old woman who was diagnosed with common variable immunodeficiency (CVID) at the age of 9 on a background of recurrent sinopulmonary infections, right inguinal lymphadenitis and her father also having CVID. Genetic testing is currently in process with results not yet available. Her other medical history was notable for exercise-induced asthma in childhood, a previous inguinal hernia repair and a remote appendectomy. She was initially treated with intravenous immunoglobulin (IVIG) until the age of 17 but began to experience significant flu-like symptoms and as result was switched to subcutaneous immunoglobulin (ScIg) therapy. Unfortunately, she had a serious reaction to ScIg consisting of syncope and seizure-like activity which led to discontinuation. She did not revert back to IVIG at the time as she was wary of experiencing side-effects and also could not be compliant. She restarted ScIg in 2020 and had been stable on a dose of 0.14 g/kg qweekly which was increased to 0.15 g/kg qweekly in December of 20,201 due to weight gain during her pregnancy. Her immunoglobulin levels from the end of November 2021 indicated an IgG 7.03 g/L (reference 6–16 g/L), IgA < 0.10 g/L (reference 0.54–4.17 g/L) and IgM 0.25 g/L (reference 0.30–2.30 g/L). These levels have been stable since she restarted ScIg in 2020. She has had good compliance with ScIg and has not missed any doses (Table 1).

**Table 1:** Patient characteristics receiving sotrovimab for treatment of mild to moderate COVID-19 infection

Identification	Medical history	Immunodeficiency history and treatment	Clinical presentation	Indication for monoclonal antibody treatment	Dose/route/frequency	Clinical effects
31-year-old 24 weeks pregnant G3P2 woman	**•** CVID • Inguinal hernia repair • Appendectomy • Right inguinal lymphadenitis	• Diagnosed at the age of 9 in the context of recurrent sinopulmonary infections • Initial blood work at time of diagnosis: - IgG 5.1 (ref 5.7–14.7 g/L) - IgA < 0.1 (ref 0.3–3.1 g/L) - IgM 0.4 (ref 0.3–2.1 g/L) - IgG1 = 4.18 g/L (ref 3.82–9.29 g/L) - IgG2 = 0.10 g/L (2.42–7.00 g/L) - IgG3 = 0.20 g/L (0.22–1.76 g/) - IgG4 = undetectable (0.039–0.864 g/L) - Measles virus IgG – reactive - Mumps virus IgG – non-reactive - Rubella virus IgG – reactive •Repeat immunization with MMR vaccine at time of diagnosis: - Measles virus IgG by EIA – positive - Mumps virus IgG by EIA – negative - Rubella virus IgG by EIA – negative • Initially on IVIG until age of 17, but switched to ScIg due to flu-like side-effects encountered with IVIG Had an adverse reaction with ScIg with syncope and seizure-like activity • Due to compliance issues and concern about developing side-effects she could not revert back to IVIG at the time • Restarted on ScIg in August 2020, cutaquig 10 g (0.14 g/kg) subQ qweekly, ↑to 12 g (0.15 g/kg) subQ qweekly in December 2021 due to weight gain in pregnancy • Blood work prior to stating ScIg in March 2020: - IgG 3.91 g/L (ref 6–16 g/L), - IgA < 0.1 g/L (ref 0.54–4.17 g/L) - IgM 0.35 g/L (ref 0.30–2.30 g/L) • Blood work from November 2021 - IgG 7.03 g/L (ref 6–16 g/L), - IgA < 0.10 g/L (ref 0.54–4.17 g/L) - IgM 0.25 g/L (ref 0.30–2.30 g/L) • Blood work from February 2022 (post sotrovimab treatment): - IgG 6.77 g/L (ref 6–16 g/L), - IgA < 0.10 g/L (ref 0.54–4.17 g/L) - IgM 0.23 g/L (ref 0.30–2.30 g/L)	• She presented January 18, 2022 to the ED with a two-day history of pharyngitis that progressed to include nasal and chest congestion, non-productive cough and shortness of breath. • She was diagnosed with COVID-19 confirmed with PCR testing	• ≥ 12 years of age with mild to moderate symptomatic COVID-19 on a background of IEI • Consideration given pregnant	**•** She received a single dose of sotrovimab 500 mg IV over one hour on the same date of testing positive for SARS-CoV-2	• Post treatment her symptoms did not progress • Symptoms of pharyngitis, nasal and chest congestion, and non-productive cough resolved within two days post treatment • Shortness of breath resolved after three days post treatment • She did not require hospitalization • She went on to have an uneventful pregnancy months later at 37 weeks

*G3P2* Gravida 3 para 2, *CVID* common variable immunodeficiency, *IVIG* intravenous immunoglobulin, *ScIg* subcutaneous immunoglobulin, *SubQ* subcutaneous, *ED* emergency department, *PCR* polymerase chain reaction, *IEI* inborn errors of immunity, *IV* intravenous, *Ref* reference, *MMR* measles, mumps, rubella

She had been vaccinated with three doses of Moderna’s COVID-19 vaccine (Spikevax ®) on April 28 2021, June 5 2021 and January 8 2021, with her third dose being a booster. She tolerated the vaccines well without any issues. She initially presented to the emergency department (ED) on January 18, 2022 with a two-day history of pharyngitis that progressed to include nasal and chest congestion, non-productive cough and shortness of breath. Her vitals at the time of the ED visit were: temperature of 35 degrees Celsius, heart rate of 109 beats per minute, blood pressure 142/92 mmHg, respiratory rate 22 breaths/minute and an oxygen saturation of 99% on room air. She was diagnosed with COVID-19 confirmed with polymerase chain reaction (PCR) testing using the BD SARS-CoV-2 (N1 + N2) assay. She believes her exposure had come from her husband who had contracted the virus, detected by rapid antigen testing at home, a few days prior after being exposed to a sick contact at work. Given that she had mild to moderate symptoms, was pregnant, had an underlying immunodeficiency and had no history of previous infection with SARS-CoV-2, she met criteria for treatment with a monoclonal antibody. As per availability, she received sotrovimab (Xevudy®) 500 mg intravenously on the same day she tested positive for the virus. After treatment, her symptoms did not progress and she did not require hospitalization. She did not receive any other treatment during her ED visit and tolerated the sotrovimab well without any issues. She was seen in follow-up by her obstetrician a few days later. She first noted symptomatic improvement of the pharyngitis, nasal and chest congestion and the non-productive cough within two days. Complete resolution of her symptoms, including the shortness of breath, was observed three days post treatment. Her immunoglobulin levels following sotrovimab treatment indicated an IgG 6.77 g/L (reference 6–16 g/L), IgA < 0.10 g/L (reference 0.54–4.17 g/L) and IgM 0.23 g/L (reference 0.30–2.30 g/L) (Table 1). Months later, she went on to have an uneventful pregnancy at 37 weeks with a healthy child.

## Discussion and conclusion

Sotrovimab is an engineered recombinant human IgG1 kappa monoclonal antibody that binds to a conserved epitope on the spike protein receptor binding domain of SARS-CoV-2 inhibiting the step after virus attachment and prior to the fusion of the viral and cell membranes. It was derived from an antibody isolated from a patient who had recovered from SARS-CoV-1 infection. In a phase 3 multicenter randomized controlled trial, 583 non-hospitalized patients with symptomatic COVID-19 (≤ 5 days after the onset of symptoms) and at least one risk factor for disease progression received either a single infusion of sotrovimab at a dose of 500 mg or placebo. These risk factors included age ≥ 55 or having underlying conditions like diabetes, chronic kidney disease, heart failure, chronic obstructive lung disease, moderate to severe asthma or obesity. Of note, patients with severe COVID-19, defined as shortness of breath at rest, an oxygen saturation below 94%, or the use of supplemental oxygen, were excluded. Furthermore, patients who were pregnant or breastfeeding, severely immunocompromised, and those who received a COVID-19 vaccine at any point prior to enrollment were excluded. Among the high-risk patients with symptomatic mild to moderate COVD-19 infection, hospitalization longer than 24 h or death was reduced by 85% with sotrovimab without an increase in adverse events. Among patients who were hospitalized, none of the patients who received sotrovimab were admitted to the ICU, as compared with five patients treated with placebo, demonstrating that sotrovimab is capable of preventing more severe complications of COVID-19 and ultimately hospitalization [4].

To this date, there have been no clinical trials evaluating sotrovimab in pregnancy or IEI. However, given its mechanism of action and demonstrated safety profile of other similar products such as IVIG and plasma in pregnant or lactating women, it is thought to be safe. As pregnancy and IEI are considered conditions where there is a higher risk of severe illness from SARS-CoV-2 infection, the benefit of treatment with sotrovimab was thought to outweigh the risks of complications associated with severe SARS-CoV-2 infection. Presently, the indications for use of sotrovimab in Canada does include IEI with consideration of use in those that are pregnant irrespective of having an underlying IEI [5].

Although further studies are needed, our novel case highlights the role of sotrovimab as a safe and potentially efficacious option for treating and preventing progression of mild to moderate symptomatic COVID-19 infection in this particular pregnant patient with CVID. This is especially important at a time when there are limited effective treatment options for patients with symptomatic COVID-19 at higher risk of disease progression. In conclusion, to our knowledge, this is the first case documenting use of a monoclonal antibody, sotrovimab, in the treatment of COVID-19 in a pregnant patient with underlying CVID.

## Data Availability

Not applicable.

## References

[1] McCusker C, Warrington R (2011). Primary immunodeficiency. Allergy, Asthma & Clin Immunol.

[2] Shields AM, Burns SO, Savic S, Richter AG, Anantharachagan A, Arumugakani G, Baker K, Bahal S, Bermingham W, Bhole M, Boules E (2021). COVID-19 in patients with primary and secondary immunodeficiency: The United Kingdom experience. J Allergy Clin Immunol.

[3] Allotey J, Stallings E, Bonet M, Yap M, Chatterjee S, Kew T, Debenham L, Llavall AC, Dixit A, Zhou D, Balaji R (2020). Clinical manifestations, risk factors, and maternal and perinatal outcomes of coronavirus disease 2019 in pregnancy: living systematic review and meta-analysis. Bmj.

[4] Gupta A, Gonzalez-Rojas Y, Juarez E, Crespo Casal M, Moya J, Falci DR, Sarkis E, Solis J, Zheng H, Scott N, Cathcart AL (2021). Early treatment for Covid-19 with SARS-CoV-2 neutralizing antibody sotrovimab. N Engl J Med.

[5] Bailey JJ, Morris AM, Bean S (2021). Evidence-based recommendations on the use of anti-SARS-CoV-2 monoclonal antibodies (casirivimab + imdevimab, and sotrovimab) for adults in Ontario. Sci Br Ont COVID-19 Sci Advis Table.

